# Targeted sequencing from cerebrospinal fluid for rapid identification of drug-resistant tuberculous meningitis

**DOI:** 10.1128/jcm.01287-23

**Published:** 2024-03-11

**Authors:** Trinh Thi Bich Tram, Le Pham Tien Trieu, Le Thanh Hoang Nhat, Do Dang Anh Thu, Nguyen Le Quang, Nguyen Duc Bang, Tran Thi Hong Chau, Guy E. Thwaites, Timothy M. Walker, Vu Thi Ngoc Ha, Nguyen Thuy Thuong Thuong

**Affiliations:** 1 Oxford University Clinical Research Unit, Ho Chi Minh City, Vietnam; 2 Pham Ngoc Thach Hospital for Tuberculosis and Lung Disease, Ho Chi Minh City, Vietnam; 3 Centre for Tropical Medicine and Global Health, Nuffield Department of Medicine, University of Oxford, Oxford, United Kingdom; University of Manitoba, Winnipeg, Canada

**Keywords:** targeted next generation sequencing, drug resistance, CSF, Deeplex

## Abstract

Mortality from tuberculous meningitis (TBM) remains around 30%, with most deaths occurring within 2 months of starting treatment. Mortality from drug-resistant strains is higher still, making early detection of drug resistance (DR) essential. Targeted next-generation sequencing (tNGS) produces high read depths, allowing the detection of DR-associated alleles with low frequencies. We applied Deeplex Myc-TB—a tNGS assay—to cerebrospinal fluid (CSF) samples from 72 adults with microbiologically confirmed TBM and compared its genomic drug susceptibility predictions to a composite reference standard of phenotypic susceptibility testing (pDST) and whole genome sequencing, as well as to clinical outcomes. Deeplex detected *Mycobacterium tuberculosis* complex DNA in 24/72 (33.3%) CSF samples and generated full DR reports for 22/24 (91.7%). The read depth generated by Deeplex correlated with semi-quantitative results from MTB/RIF Xpert. Alleles with <20% frequency were seen at canonical loci associated with first-line DR. Disregarding these low-frequency alleles, Deeplex had 100% concordance with the composite reference standard for all drugs except pyrazinamide and streptomycin. Three patients had positive CSF cultures after 30 days of treatment; reference tests and Deeplex identified isoniazid resistance in two, and Deeplex alone identified low-frequency rifampin resistance alleles in one. Five patients died, of whom one had pDST-identified pyrazinamide resistance. tNGS on CSF can rapidly and accurately detect drug-resistant TBM, but its application is limited to those with higher bacterial loads. In those with lower bacterial burdens, alternative approaches need to be developed for both diagnosis and resistance detection.

## INTRODUCTION

Tuberculosis meningitis (TBM) is caused by the hematogenous spread of *Mycobacterium tuberculosis* (*Mtb*) to the brain. It is the most severe and fatal form of tuberculosis. The incidence of TBM varies between 0.3% and 4.9% of all people with tuberculosis, translating to between 30,000 and 490,000 people with TBM in the world each year (1, 2). Despite appropriate anti-tuberculosis treatment, the disease has around 30% mortality, increasing to 50% among those who are co-infected with HIV (3). Most deaths occur within 2 months of starting treatment.

Isoniazid and rifampin remain the backbone of TBM treatment for most patients. However, identifying those patients with rifampin and isoniazid resistance is even more important in TBM than in other forms of the disease as the mortality is high without the right treatment (4). Isoniazid mono-resistance and rifampin resistance are seen in increasing numbers of TBM patients in many countries, ranging from 10% to 26% and 2% to 18%, respectively (5–8). Mortality from multi-drug resistant (MDR)-TBM approaches almost 100% in the absence of an appropriate therapy but is 40%–60% even when appropriate second-line regimens are used (5, 6, 9). Without alternative anti-tuberculosis therapy, isoniazid mono-resistance is also associated with increased morbidity and mortality (7). Early detection of drug resistance could therefore greatly improve clinical outcomes for patients with TBM.

In July 2023, the World Health Organization (WHO) recommended the use of targeted next-generation sequencing (tNGS) for drug susceptibility testing (10). This relatively new diagnostic approach works by amplifying target genes in the *Mtb* genome and sequencing the amplicons to a great depth on either short- or long-read sequencing platforms. Much remains to be learned about the clinical significance of low-frequency alleles that are likely to be detected through deep sequencing.

Among the three commercial targeted sequencing assays recommended by WHO, only Deeplex Myc-TB from GenoScreen (referred as Deeplex) can detect the resistance up to 15 anti-tuberculosis drugs including bedaquiline used in a novel all-oral 6-month regimen for MDR tuberculosis. This targeted sequencing assay covers 18 gene regions associated with drug resistance and identification of the *Mtb* complex (11). It has shown early promise in the detection of drug resistance directly from sputum samples, although the performance has been better for samples with higher smear microscopy grades (11, 12). The use of Deeplex for non-sputum samples that often have lower bacterial load is less well-studied.

Here, we use the Deeplex tNGS platform on an archived collection of cerebrospinal fluid (CSF) samples from adults recruited to recent TBM clinical trials conducted in Vietnam. We compare the performance of Deeplex to a composite standard of culture-based phenotypic drug susceptibility tests (pDSTs) and whole genome sequencing (WGS) for detecting drug resistance and explore how previously undetected resistant alleles might have had an impact on clinical outcomes.

## MATERIALS AND METHODS

### Participants

Adults (≥18 years) with TBM were recruited from a clinical trial conducted at Pham Ngoc Thach Hospital and the Hospital of Tropical Diseases, Ho Chi Minh city from June 2011 to March 2015 (13). The patients had meningitis symptoms, which included headache, nuchal rigidity, abnormal cerebrospinal fluid parameters (including color, opening pressure, white blood cell count, protein, lactate, and glucose), and acid fast bacilli seen in the cerebrospinal fluid by Ziehl-Neelsen stain or *Mtb* isolated by culture. Written informed consent was obtained from all participants or their relatives if they were incapacitated, prior to study entry and sample collection.

### Samples

As part of the trial, 5–10 mL of CSF was obtained from all patients, concentrated by centrifugation at 3000 × *g* for 15 minutes, and re-suspended in 700 µL of CSF supernatant (a buffer solution would have sufficed too). This CSF deposit was used for the diagnostic tests, including 100 µL for Ziehl-Neelsen smear, 200 µL for Xpert MTB/RIF, and 200 µL for mycobacterial growth indicator tube (MGIT) culture. The remaining 200 µL of CSF was stored at −80°C, from which 117 samples were used for molecular bacterial load assay to rapidly quantify viable *Mtb* using 16S rRNA gene sequencing (14). This study made use of the remaining samples of stored CSF deposit.

**Fig 1:**
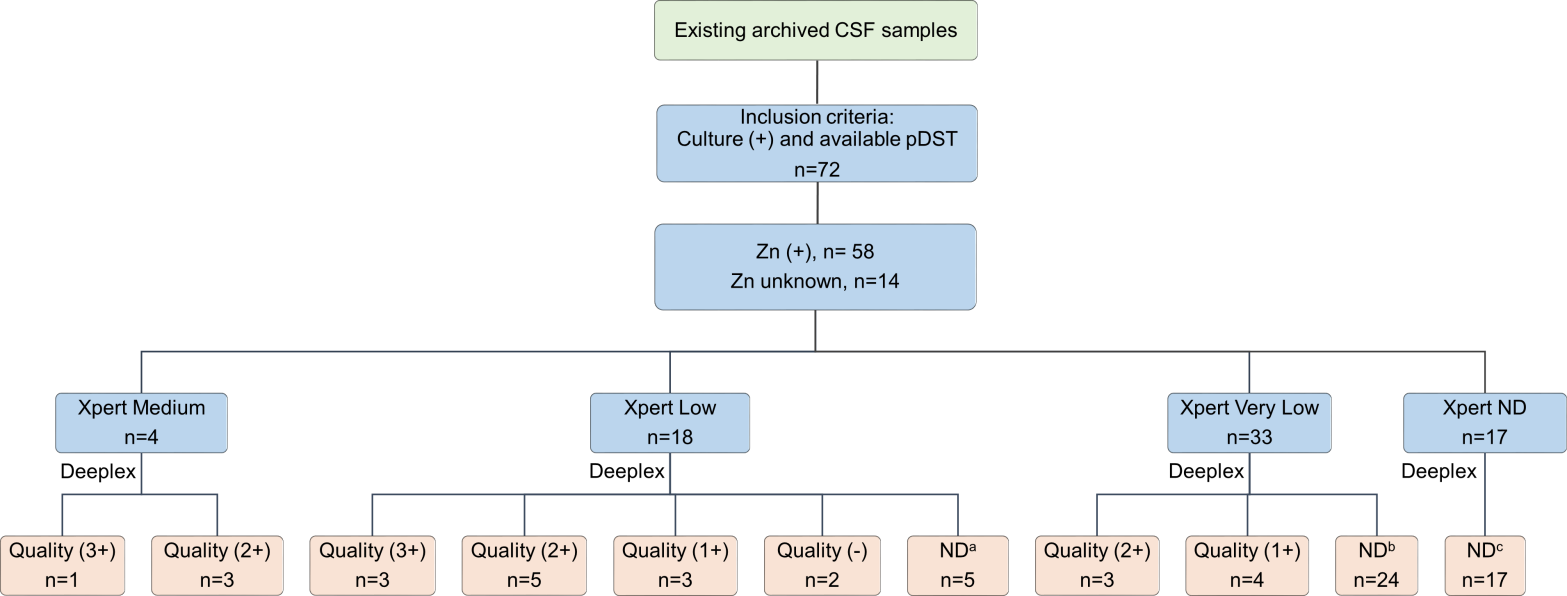
Study flow chart demonstrating the results of Deeplex (in orange) in relation to microbial test results (in light blue). Deeplex sequencing quality was classified as: not detected (ND) for mycobacteria, – for partial reads, 1+, 2+, and 3 + for complete reads with adequate depth of coverage. Zn: Ziehl-Neelsen stain, ^a^1 sample with Zn not done, ^b^5 samples with Zn not done, and ^c^8 samples with Zn not done.

The number of CSF samples required for the study was calculated following the formula for diagnostic studies with binary test outcome (here resistance or susceptible) (15). Deeplex can detect *Mtb* in 80.0% of culture-positive sputum samples, with high sensitivity (95.0%) and specificity (97.0%) for detecting *Mtb* drug resistance (11, 12, 16, 17). Therefore, around 43 CSF samples, including at least 29 resistant to any first-line drug or streptomycin and 14 fully susceptible samples, would be required to detect a sensitivity and specificity of 95.0% with the marginal errors for sensitivity and specificity of 8.0% and 11.0%, respectively. Deeplex has been reported to predict the resistance phenotype in ~60.0%–70.0% of clinical samples with a microscopy grading of 0 or 1 (11). We thus assume that only 60.0% of culture-positive CSF samples can be detected by Deeplex due to the low bacillary burden. This then translates into 72 required samples (47 resistant and 25 susceptible). Therefore, 72 CSF samples were included in the study, archived from 72 patients who had culture-confirmed TBM and available pDST by MGIT for first-line drugs (Fig. 1).

### Bacteriological test report

Ziehl-Neelsen stain was reported as negative or positive with *Mtb* without grading. Interpretation of GeneXpert MTB/RIF result was performed with GeneXpert software version 4.0b. Semi-quantitative mycobacterial load results were reported as follows: very low [cycle threshold (Ct)  > 28], low (Ct 22–28), medium (Ct 16–22), or high (Ct  < 16) (18). Ct values for each of the five probes were recorded. Xpert Ct-value for statistical analysis as described below was calculated by the average Ct of five probes with value >0. For culture by BD Bactec MGIT 960 instrument, the time to culture positivity by days for each sample was recorded.

### *Mtb* DNA extraction

Frozen CSF samples were thawed and heat inactivated on a thermal block for 30 minutes at 80°C. DNA was then extracted using a previously described mechanical disruption method (19). Briefly, samples were incubated with 4M guanidine thiocyanate (GTC) lysis buffer (Sigma, USA) to denature the membrane protein of eukaryotic and Gram-negative cells. After being washed and re-suspended in 100 µL of water, samples were then subjected to three rounds of bead-beating at 6 m/s for 40 seconds. The beads were pelleted by centrifugation at 13,000 × *g* for 10 minutes, and 50 µL of supernatant was cleaned with 90 µL of AMPure beads (Beckman Coulter, UK). Samples were eluted in 25 µL of water and quantified with a Qubit fluorometer (Thermo Fisher Scientific, USA).

### Deeplex and whole genome sequencing

tNGS on DNA from CSF by Deeplex assay was performed according to the GenoScreen Deeplex Myc-TB user manual (20). The assay targets full sequences (i.e., coding sequence plus part of promoter region) or the most relevant regions of 18 genes associated with resistance to 13 anti-TB drugs rifampin (*rpoB*), isoniazid (*inhA, fabG1, katG and ahpC*), pyrazinamide (*pncA*), ethambutol (*embB*), streptomycin (*rrs, gidB, rpsL*), kanamycin (*rrs, eis*), amikacin (*rrs*), capreomycin (rrs, tlyA), fluoroquinolones (*gyrA, gyrB*), ethionamide (*ethA, inhA, fabG1*), linezolid (*rplC, rrl*), bedaquiline and clofazimine (*Rv0678*), combined with genomic targets for mycobacterial species identification (*hsp65*) and *Mtb* complex strain genotyping (spoligotype). Briefly, 9 µL of DNA extract was amplified by 24-plex PCR using a Mastermix. Amplicon libraries were prepared using commercially available kits (Nextera XT DNA Library Prep Kit) following the manufacturer’s instructions. Batches of 48 samples were sequenced on an Illumina MiSeq sequencer using Illumina MiSeq V2 to generate 150 base pair paired-end reads.

For WGS, *Mtb* DNA from each isolate was used to prepare a library using the Nextera XT DNA Library Prep Kit. All libraries were sequenced in 2 × 150 bp Illumina MiSeq run sequencing using MiSeq V2 reagent kits (Illumina, USA), multiplexing 18 samples per run.

### Sequencing analysis

FASTQ data generated on the Illumina MiSeq machine were uploaded directly to Deeplex web application for automatic analysis of species identification and drug susceptibility. Mycobacterial species were first identified based on the nucleotide identity of the *hsp65* gene. *Mtb* strains were then *in silico* spoligotyped and genotyped by direct repeat regions and phylogenetic single-nucleotide polymorphisms (SNPs), respectively. Alignment to *Mtb* H37Rv reference sequences was performed using Bowtie 2, and variants were called with a limit of 3% read proportion depending on the depth of targets. Samples were then classified in accordance with the breadth of target coverage and categorized by quality as: ND (mycobacteria not detected), − (at least one resistance-associated position not covered by reads), 1+ (resistance-associated positions in the database can be identified with an allele frequency of ≥80.0%) , 2+ (resistance-associated positions in the database can be identified with an allele frequency of ≥10.0%), or 3+ (resistance-associated positions in the database can be identified with an allele frequency of ≥3.0%). Detected variants were compared with an inbuilt Deeplex reference database of mutations associated with drug-resistance and lineage; resistance was reported if any drug resistance-associated variant was detected at any allele frequency. Variants not included in the database were defined as “uncharacterized” by Deeplex and no prediction was made unless another resistance-associated mutation was also present.

For WGS, FASTQ data generated on the Illumina MiSeq machine were trimmed using *bbduk*, mapped against the H37Rv reference genome (NC_000962.3) using *bwa mem* (21), and SNPs were called using GATK (version 3.8–1–0-gf15c1c3ef) in unified genotyper mode (22). These steps were executed by the PHEnix pipeline (https://github.com/phe-bioinformatics/PHEnix). *Mtb* complex and antibiotic resistance to isoniazid, rifampin, ethambutol, pyrazinamide, streptomycin, amikacin, and moxifloxacin were identified and predicted from WGS using Mykrobe predictor software v0.10.0 (https://github.com/Mykrobe-tools/mykrobe). Drug resistance was called where resistance-conferring mutations were present at an allele frequency ≥90.0% (23).

### Reference standards

For 72 CSF samples from which *Mtb* had previously been cultured, pDST was performed in MGIT for isoniazid, rifampin, ethambutol, pyrazinamide, and streptomycin. WGS was performed for all isolates, meaning that for second-line drugs only WGS-based DST results were available. Where the results of both pDST and WGS were available, a composite reference standard of pDST and WGS was used. This was helpful as resistance mutations that elevate the MIC only marginally over the critical concentration can be missed by pDST alone. Drug resistance was thereby defined as a resistant result from either pDST or WGS, or both. An isolate was considered drug susceptible when both pDST and WGS were susceptible.

### Statistical analysis

To assess the association between *Mtb* detection by Deeplex and the bacterial load quantified by Xpert Ct-value or time to MGIT culture positivity, we used a logistic regression model including *Mtb* detection as an outcome and Xpert Ct-value or time to positivity as a covariate. We modeled the non-linear trend of time to positivity using a natural cubic spline model with boundary knots of 0 and 25 and inner knots of 10 and 15. We chose the linear trend or non-linear association of Ct-value or time to positivity based on Likelihood ratio test. Figures were generated in R program v4.0.2 (24).

## RESULTS

### Performance of Deeplex

One CSF sample from each of the 72 patients with TBM was selected from an archive of clinical trial samples. Among these, smear microscopy was originally positive in 58/72 samples and not performed for 14/72 (Fig. 1). Xpert MTB/RIF detected *Mtb* in 76% (55/72) (Table 1), and rifampin resistance in 6 (Table S1). Overall, Deeplex detected *Mtb* complex DNA in 24/72 (33.3%), of which 22 were sufficient reads for drug susceptibility predictions for all 13 anti-tuberculosis drugs (Table 1). All 24 were Xpert RIF-sensitive (Table S1).

**TABLE 1:** Performance of Deeplex in CSF samples (*n* = 72) for the detection of *Mtb* and *Mtb* drug resistance

Xpert MTB/RIF	Detection of *Mtb* complex (n, %)	Reads qualified for all 13 drugs
Medium	4/4 (100)	4/4 (100)
Low	13/18 (72.2)	11/13 (84.6)
Very low	7/33 (23.5)	7/7 (100)
Not detected	0/17 (0.0)	0/0 (0.0)
Overall (n, %)	24/72 (33.3)	22/24 (91.7)

**Fig 2:**
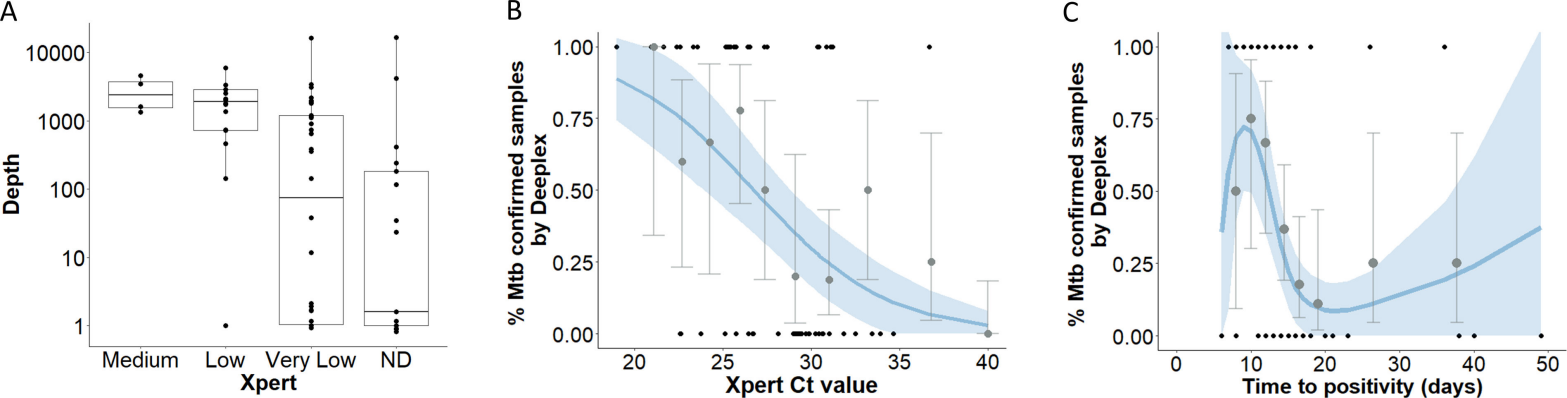
Performance of Deeplex in *Mtb* detection in relation to other microbiological confirmed tests. (**A**) Mean read depth of CSF samples by Deeplex against semi-quantitative Xpert result. Box plots represent its median and 1st/3rd interquartile and dots represent individual samples. ND: Not detected. (**B and C**) Association between Deeplex *Mtb* detection and Xpert Ct-value readouts (**B**) or time to culture positivity (**C**) by logistic regression model. Black dots show the observed values. Gray dots and error bars show the mean values and 95% CI of observed quantiles. Blue line shows the predicted values from logistic regression model and the shaded area shows the 95% CI of predicted values.

The quality of Deeplex results increased with bacterial load by Xpert (Fig. 1). The median depth of reads generated by Deeplex varied by Xpert-derived semi-quantitative bacterial load (2546 for Xpert medium, 1900 for low, and <100 for very low or undetected) (Fig. 2A; Table S1). There was a relationship between bacterial load by Xpert and the sensitivity of Deeplex for detecting *Mtb*. The assay detected *Mtb* complex in 4/4 (100%) with a semi-quantitative bacterial load of medium; 13/18 (72.2%) where it was low; 7/33 (21.2%) where it was very low; and 0/17 where *Mtb* was undetected by Xpert MTB/RIF (Table 1). This relationship was also confirmed with a negative association between Deeplex *Mtb* detection and Xpert Ct values by a logistic regression model [odds ratio (OR) (95% confidence interval (CI)) = 0.77 (0.66–0.89), *P*-value = 0.0003] (Fig. 2B), whereas the relationship between *Mtb* detection by Deeplex and the time to culture positivity was non-linear (*P*-value = 0.005) (Fig. 2C; Table S2).

### Resistance detected by Deeplex

**Fig 3:**
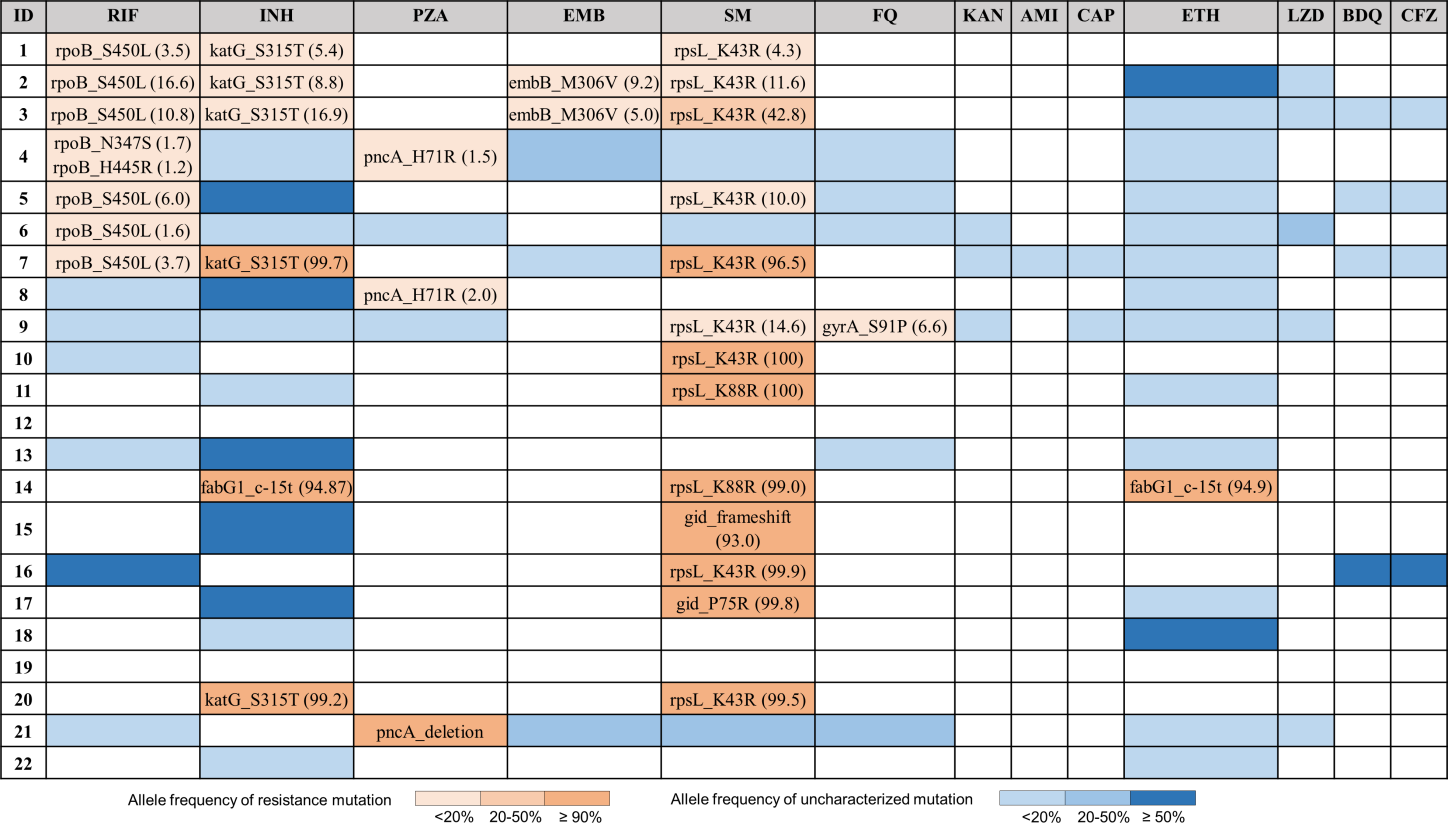
Extent of drug-resistant mutations in 22 CSF samples by Deeplex. Mutations associated with drug resistance are specified in the cells when present. Numbers in the brackets indicate the proportions of reads carrying resistant allele. RIF, rifampin; INH, isoniazid; PZA, pyrazinamide; EMB, ethambutol; SM, streptomycin; FQ, fluoroquinolones; KAN, kanamycin; AMI, amikacin; CAP, capreomycin; ETH, ethionamide ; LZD, linezolid; BDQ, bedaquiline; and CFZ, clofazimine. A dark to light orange gradient represents for resistance mutations with allele frequencies indicated by color, while a dark to light blue gradient represents for uncharacterized mutations with allele frequencies indicated by color.

There was a bimodal distribution of allele frequency among detected drug resistance variants, with 12 having an allele frequency ≥90.0% and 19 with an allele frequency <20.0% (Fig. 3). Only one isolate had a higher-frequency minority allele, relevant to streptomycin, at 43.0%. As the sample size was overall small, we did not further discriminate between allele frequencies of 3.0% and 20.0% when presenting our findings below. In one sample, the Deeplex results indicated that the whole of *pncA* was deleted, but on the inspection of the WGS bam file of reads mapped to the reference H37Rv, the gene was present, suggesting an amplification error in the Deeplex workflow. While the fixed variants were seen in genes relevant to isoniazid (*fabG1*_c-15t and *katG*_S315T) and streptomycin (*gid*_P75R, *rpsL*_K43R, *rpsL*_K88R), drugs to which resistance is most commonly seen in this context, hetero-resistance (the presence of resistance mutations at an allele frequency <90.0%) was seen at canonical resistance loci across genes relevant to first-line drugs, streptomycin and fluoroquinolones (*katG*_S315T, *rpoB*_S450L, *rpoB*_N347S, *rpoB*_H445R, *embB*_M306V, *pncA* H71R*, rpsL*_K43R, and *gyrA*_S91P) (Fig. 3). A number of uncharacterized variants that have yet unknown association with drug resistance were also commonly seen across genes relevant to both first- and second-line drugs (Table S3).

To assess the accuracy of Deeplex’s drug susceptibility predictions based on fixed variants, and to assess the significance of low-frequency alleles at drug resistance loci, comparison was made to results obtained from WGS and pDST (Table 2). Where both were available, a composite of the two (pDST and WGS) was used (Table S4). The discordance between Deeplex and composite reference standard was observed for samples with minority variants. All samples with minority alleles (<20.0%) at resistance loci relevant to first-line drugs (rifampin, isoniazid, ethambutol, and pyrazinamide) were susceptible by the composite reference standard. One sample with a minority allele *gyrA*_S91P (6.6%) was susceptible by WGS to fluoroquinolones (no pDST result available), and one sample with a minority allele *rpsL*_K43R at a frequency of 43% was resistant by both pDST and WGS (Fig. 3; Table 2). Disregarding minority alleles (<20.0%), all results were concordant with the exception of two results for pyrazinamide and one for streptomycin (Table 2).

**TABLE 2:** Concordance of drug resistance detection by Deeplex, WGS, and MGIT pDST^*c*^

	Resistant by composite references (WGS and pDST)			Susceptible by composite references (WGS and pDST)				
Drugs	Resistant by Deeplex	Resistant (minority allele) by Deeplex	Susceptible by Deeplex	Resistant by Deeplex	Resistant (minority allele) by Deeplex	Susceptible by Deeplex	Resistant alleles with any frequency (%)	Resistant alleles with ≥20.0% frequency (%)
RIF	0	0	0	0	7	15	15/22 (68.2)	22/22 (100)
INH	3	0	0	0	3	16	19/22 (86.4)	22/22 (100)
PZA	0	0	1	1	2	18	18/22 (81.8)	20/22 (90.9)
EMB	0	0	0	0	2	20	20/22 (90.9)	22/22 (100)
SM	7	1^*b*^	0	1	4	9	17/22 (77.3)	21/22 (94.5)
FQ	0	0	0	0	1	21	21/22 (95.4)	22/22 (100)
KAN^*a*^	0	0	0	0	0	22	22/22 (100)	22/22 (100)
AMI^*a*^	0	0	0	0	0	22	22/22 (100)	22/22 (100)
CAP^*a*^	0	0	0	0	0	22	22/22 (100)	22/22 (100)
ETH^*a*^	1	0	0	0	0	21	22/22 (100)	22/22 (100)
LNZ^*a*^	0	0	0	0	0	22	22/22 (100)	22/22 (100)
BDQ^*a*^	0	0	0	0	0	22	22/22 (100)	22/22 (100)
CFZ^*a*^	0	0	0	0	0	22	22/22 (100)	22/22 (100)

^*a*^ Genotypic DST from WGS was used as a standard reference.

^*b*^ Resistant allele frequency of 43.0%.

^*c*^ RIF, rifampin; INH, isoniazid; PZA, pyrazinamide; EMB, ethambutol; SM, streptomycin; FQ, fluoroquinolones; KAN, kanamycin; AMI, amikacin; CAP, capreomycin; ETH, ethionamide; LNZ, linezolid; BDQ, bedaquiline; and CFZ, clofazimine.

### Microbiological and clinical treatment responses

All 22 patients who had susceptibility predictions for all 13 anti-TB drugs by Deeplex were treated with first-line drugs (Table 3). Sterile CSF cultures were obtained at day 30 of treatment in 17 (77.2%) patients: one had isoniazid resistance by both Deeplex and composite reference, one had pyrazinamide resistance by only Deeplex, eight were pan-susceptible to first-line drugs by both Deeplex and the composite reference, and seven were hetero-resistant to at least one first-line drug by Deeplex. Positive CSF MGIT cultures after 30 days of treatment were obtained from three patients, for whom Deeplex and reference tests identified one sample as susceptible to all first-line drugs and two as isoniazid resistant. For one of the isoniazid resistant samples, Deeplex also identified a low-frequency (<20.0%) allele at a locus associated with rifampin resistance. Two patients died within 30 days of hospitalization, three died at a later stage, 15 were alive after 9 months of treatment, and two were lost to follow-up (Table 3). Pyrazinamide resistance was detected by MGIT, but not by WGS or Deeplex, for one patient who died. All other signals for drug resistance were seen exclusively in patients with disease free survival.

**TABLE 3:** Time to culture conversion and treatment outcome of TBM patients whose drug susceptibility predictions by Deeplex were successful^*c*^

ID	First-line drug resistance by composite reference	First-line drug resistance by Deeplex	First-line hetero-resistance by Deeplex^*a*^	Standard treatment regimen	Time to culture conversion (days)	Treatment outcome after 9-month treatment
1			R, H	3RHZE/6RH	30	Alive
2			R, H, E	3RHZE/6RH	30	Alive
3			R, H, E	3RHZE/6RH	30	Alive
4			R, Z	3RHZE/6RH	30	Alive
5			R	3RHZE/6RH	30	Alive
6			R	3RHZE/6RH	30	Alive
7	H	H	R	3RHZE/6RH	60	Alive
8			Z	3RHZE/6RH	30	Alive
9				3RHZE/6RH	30	Lost to follow-up
10				3RHZE/6RH	30	Died
11				3RHZE/6RH	30	Lost to follow-up
12				3RHZE/6RH	30	Alive
13				3RHZE/6RH	30	Alive
14	H	H		3RHZE/6RH	30	Alive
15				3RHZE/6RH	30	Died
16				3RHZE/6RH	**60**	Alive
17				3RHZE/6RH	30	Died
18	Z^*b*^			3RHZE/6RH	0	Died within 30 days
19				3RHZE/6RH	30	Alive
20	H	H		3RHZE/6RH	60	Alive
21		Z		3RHZE/6RH	30	Alive
22				3RHZE/6RH	0	Died within 30 days

^*a*^ The frequency of minority alleles at loci relevant to drug resistance was <20%.

^*b*^ Pyrazinamide resistance was detected by pDST but not WGS.

^*c*^ R, rifampin; H, isoniazid; Z, pyrazinamide; and E, ethambutol.

Deeplex also provided data on spoligotype and SNP-based phylogenetic lineage classification. SNP-based lineage prediction was possible for 24/72 samples (Table S5), of which two were reported to contain two lineages, and seven were reported to contain more than two lineages. Although 6/9 samples with an apparent mixture of lineages also had hetero-resistance, the more lineages reported in a sample, the more likely this was due to artifact. WGS did not identify the evidence of more than one lineage in any of the corresponding isolates.

## DISCUSSION

Rapid detection of drug resistance could enable prompt appropriate treatment of TBM, thereby reducing mortality and morbidity. tNGS is currently recommended by WHO for its use on primary clinical samples in those with pulmonary tuberculosis, with the potential for faster turn-around time to results than culture-based pDST. Deeplex has previously generated accurate DST predictions from sputum samples and met the class-based performance by WHO (11, 12). Here, we demonstrate its potential utility when applied to CSF samples from adults with TBM.

Our results are consistent with previous findings that both culture and Xpert MTB/RIF are more sensitive than Deeplex for samples with low bacillary burdens (16). Deeplex failed to detect *Mtb* in any of the 17 samples that were Xpert negative but culture positive. Its performance improved as the semi-quantitative results or Ct-value readouts from Xpert MTB/RIF increased. However, when *Mtb* was detected by Deeplex, drug susceptibility could be predicted accurately for the vast majority of cases. It thus appears that a preliminary quantitative bacterial load assay may have a role in determining which samples contain sufficient *Mtb* to warrant the early use of Deeplex to detect drug resistance.

tNGS from direct samples has potential to greatly accelerate the time to results. As demonstrated elsewhere, it takes 32–71 days to get results by pDST or 22–58 days for WGS (including 20–56 days of culture positive plus 2 days of sequencing), while tNGS takes just 2–3 days (16). Another potential advantage of tNGS is the opportunity to detect hetero-resistance from direct clinical samples. The need to better understand the clinical relevance of hetero-resistance at different allele frequencies will grow as these new assays are used more. It could for example have a major impact on the outcome of therapy. Xpert MTB/RIF detects rifampin mutations that are present in at least 20.0% of the mixture (25), whereas studies using line probe assay (LPA) on culture from CSF have reported hetero-resistance for isoniazid and rifampin at frequencies of 4.0% and 1.0%, respectively (26). Deeplex can detect hetero-resistance down to 3.0% sub-populations and possibly still lower (27). We detected hetero-resistance to those drugs we would commonly expect to see drug resistance to and at canonical resistance loci rather than rarer ones. It is thus highly likely that Deeplex is detecting the actual emergence of resistant populations and not mere noise from stray reads (28). However, Deeplex also detected more than one lineage in 6/9 CSF samples with hetero-resistance, suggesting that the hetero-resistance may also be due to a mixed infection of resistant and susceptible strains.

According to the manufacturer’s instructions, resistant mutations at any allele frequency should be interpreted as predictive of resistance to the relevant drug. It is hard to justify this evidentially given that such minority populations are often lost during culture. The discordance we observe between Deeplex and culture-based WGS and pDST when we take low-frequency alleles into account is thus to be expected. However, when one disregards such low-frequency alleles (<20.0%), we see extremely high concordance between Deeplex and the composite reference. The small signal we do see from our exploratory data in terms of delayed culture-conversion may be indicative of an impact of hetero-resistance, even though no impact on final clinical outcome was observed. As tNGS is now WHO recommended, the significance of hetero-resistance is likely to become an important question given how common it seems to be in some data sets. To use tNGS as a test that informs treatment choices, we will need to better understand how common the hetero-resistances is, which hetero-mutations we need to be concerned about, and which not, and at which frequency. Without such understanding, we risk overcalling resistance and potentially withholding effective drugs from patients. Our data raise some questions but much larger data sets are required to answer them.

The prediction of pyrazinamide resistance is always challenging, and concordance between pDST and WGS can be poor (29). Unsurprisingly, we observed one case that was indicated as resistant in MGIT but predicted to be susceptible by either Deeplex or WGS. However, we also encountered a sample for which Deeplex reported pyrazinamide resistance due to the apparent whole-gene deletion of *pncA*. As the strain was susceptible by pDST and WGS, which detected *pncA* as present, we inferred an amplification failure on the part of Deeplex. Such technical issues need to be borne in mind, and any large deletions detected by Deeplex should be scrutinized carefully when interpreting results. Another issue we observed was that Deeplex reported an infection with a mixture of lineages on multiple occasions. While in some instances there may have been genuine mixed infections, in others, for example, where a mixture of lineage 1, *Mycobacterium africanum*, and *Mycobacterium canettii* was reported, this was most likely due to artifacts as neither *M. africanum* nor *M. canettii* are seen in Vietnam.

The actual cost of tNGS by Deeplex is currently higher than culture-based WGS (e.g., 204 USD vs. 150 USD by local price). However, tNGS has been shown to be cost-effective in a model-based cost-effectiveness analysis for WHO, when positioned either as an initial DST test or as a reflex test in the case of rifampin resistance (10). Even greater cost-effectiveness could be seen if earlier effective treatment is achieved.

There are a number of limitations to our study. The sample size was small and the study design was retrospective, although this also enabled us to look at the potential impact of hetero-resistance on clinical outcome. We did not have pDST results for all drugs for all samples; hence, we could not assess the nature of some of the putative drug-resistant minority variants. While we performed WGS on cultured isolates, we did not confirm the presence of low-frequency alleles detected by Deeplex in clinical samples through an independent method applied directly to those clinical samples. A number of mutations detected in our data were of uncertain significance in relation to drug resistance due to the absence of information on these mutations in the Deeplex reference database. Given that the Deeplex’s reference database is partly informed by the WHO mutation catalogue, consistent updates to the catalogue by WHO could provide more accurate predictions in the future.

Our study suggests that tNGS by Deeplex could be a promising tool for detecting drug-resistant TBM when there are sufficient bacterial loads. The appeal of tNGS is its accuracy in detecting resistance mutations and potential for rapid turn-around time leading to targeted therapy regimens and improved outcomes for patients with TBM. Although Deeplex also provides data on minority variants, often at drug resistance loci, the clinical significance of these remains to be determined. Given that many people with TBM have bacterial loads below the threshold of tNGS and most other microbiological tests, there remains an unmet need to develop alternative diagnostic approaches for TBM and the rapid detection of drug-resistant bacteria in CSF.

## Data Availability

The sequencing data have been deposited in the European Nucleotide Archive (ENA) database (accession no. PRJEB66382).

## SUPPLEMENTAL MATERIAL

Tables S1, S2, S3, S4, and S5 jcm.01287-23-s0001.xlsx Supplemental tables.

ASM does not own the copyrights to Supplemental Material that may be linked to, or accessed through, an article. The authors have granted ASM a non-exclusive, world-wide license to publish the Supplemental Material files. Please contact the corresponding author directly for reuse.
